# Condition‐dependent ejaculate production affects male mating behavior in the common bedbug *Cimex lectularius*

**DOI:** 10.1002/ece3.2073

**Published:** 2016-03-14

**Authors:** Bettina Kaldun, Oliver Otti

**Affiliations:** ^1^ Animal Population Ecology, Animal Ecology IUniversity of BayreuthUniversitätsstrasse 3095440BayreuthGermany

**Keywords:** Feeding behavior, mating behavior, reproductive physiology, sperm ecology, sperm physiology

## Abstract

Food availability in the environment is often low and variable, constraining organisms in their resource allocation to different life‐history traits. For example, variation in food availability is likely to induce condition‐dependent investment in reproduction. Further, diet has been shown to affect ejaculate size, composition and quality. How these effects translate into male reproductive success or change male mating behavior is still largely unknown. Here, we concentrated on the effect of meal size on ejaculate production, male reproductive success and mating behavior in the common bedbug *Cimex lectularius*. We analyzed the production of sperm and seminal fluid within three different feeding regimes in six different populations. Males receiving large meals produced significantly more sperm and seminal fluid than males receiving small meals or no meals at all. While such condition‐dependent ejaculate production did not affect the number of offspring produced after a single mating, food‐restricted males could perform significantly fewer matings than fully fed males. Therefore, in a multiple mating context food‐restricted males paid a fitness cost and might have to adjust their mating strategy according to the ejaculate available to them. Our results indicate that meal size has no direct effect on ejaculate quality, but food availability forces a condition‐dependent mating rate on males. Environmental variation translating into variation in male reproductive traits reveals that natural selection can interact with sexual selection and shape reproductive traits. As males can modulate their ejaculate size depending on the mating situation, future studies are needed to elucidate whether environmental variation affecting the amount of ejaculate available might induce different mating strategies.

## Introduction

Food is an essential component in life. Organisms need to eat to be able to grow, to maintain their everyday activity and to reproduce (Boggs 2009). To optimize fitness, both the quantity and the composition of nutrients seem important for organisms (Lee et al. 2008). Although females and males often differ in their reproductive interests (Andersson 1994), for both sexes variation in food availability will impose selection on resource allocation to reproduction depending on their condition, that is depending on the resources available to them (Boggs 1992; Hodin 2009). That male secondary sexually selected traits show condition dependence is empirically well established (Cotton et al. 2004). However, the condition dependence of more primary reproductive traits in males, such as the production of ejaculate components, is less well understood. In ladybird beetles, Perry and Rowe (2010) have shown that ejaculate size and its composition are condition dependent. While sperm are relatively inexpensive, ejaculates are often costly to produce (e.g., Dewsbury 1982). The consequences of condition‐dependent ejaculate production on male fitness are largely unknown (Morehouse et al. 2010). Variation in food availability is likely to define variation in sperm number and probably also sperm function. For example, in reproductive medicine environmental factors, so‐called lifestyle effects, such as diet, smoking habits are known to alter a variety of biochemical characteristics of sperm and lead to variation in sperm function (Sofikitis et al. 1995; Jensen et al. 2004; Aitken et al. 2014). Diet has also been shown to change sperm morphology in lizards (Kahrl and Cox 2015) and the composition of the ejaculate in ladybird beetles (Perry and Rowe 2010). Further, seminal fluid influences male sperm competitive ability and some of its components manipulate the reproductive physiology of females to the male's benefit (Poiani 2006; Reinhardt et al. 2009a; Avila et al. 2011). Food availability might impact on male reproductive output by changing the amount of seminal fluid relative to sperm and/or its composition (Fricke et al. 2008; Perry and Rowe 2010). Under variable environmental conditions, males should therefore balance the use of each ejaculate component among different matings (Reinhardt et al. 2011). By affecting ejaculate size, quality or composition, variation in food availability can therefore be assumed to translate into variation in male reproductive success (Zikovitz and Agrawal 2013).

In addition, males from several species are known to adjust their ejaculate expenditure according to the mating status of the female they mate with (Simmons 2001; Sirot et al. 2011). If food restriction influences the amount of sperm and seminal fluid that can be produced, male feeding status might affect mating behavior, for example mating rate or copulation duration. Males could adjust ejaculate expenditure according to their feeding status, that is the amount of ejaculate available to a male, to optimize their mating strategy. If males are low on available ejaculate, they can adopt two different strategies: (1) use up all the available ejaculate as fast as possible reducing the number of matings thereby increasing the reproductive output per mating or (2) reduce ejaculate expenditure per mating to save on ejaculate (Wedell et al. 2002; Hodgson and Hosken 2006) to mate as many times as possible. Therefore, food restriction would either manifest in a low reproductive output per mating or a low mating rate. Besides the effect on ejaculate production, feeding might directly interfere with mating behavior, for example copulation duration. With a full belly and digestion in progress, the willingness or ability to mount a female might be reduced. Therefore, mating propensity and copulation duration might change with feeding status.

Here, we investigated whether ejaculate production is condition dependent and whether this has consequences for male mating behavior and male fitness in the common bedbug, *Cimex lectularius*. In a first step, we established if food restriction influences the production of sperm and seminal fluid. For simplicity, we focused on the effect of one feeding event on reproduction, even though bedbug males feed continuously. However, they feed very irregularly throughout their lives; therefore, it can be assumed that feeding levels vary considerably between males. Second, we investigated potential effects of food restriction on male fitness by comparing (1) the fecundity of females singly mated to males from three different feeding regimes and (2) the mating rates for males from three different feeding regimes. Third, we tested whether meal size affects mating propensity and/or copulation duration as large meals might have a negative effect on male mating ability directly after feeding.

## Material and Methods

### Biological system

Bedbugs (*C. lectularius* L.) represent a suitable model system for examining the consequences of sexual selection and reproductive physiology (Stutt and Siva‐Jothy 2001; Reinhardt et al. 2003). Male bedbugs traumatically inseminate females by piercing the female's abdominal wall with their intromittent organ (Siva‐Jothy and Stutt 2003). Most matings occur shortly after the female's bloodmeal, because fully fed females cannot resist mating (Reinhardt and Siva‐Jothy 2007). Males control the mating rate and therefore the ejaculate supply (Siva‐Jothy and Stutt 2003; Reinhardt et al. 2009b).

All bedbugs were maintained in a CT room at 26 ± 1°C, at about 70% relative humidity with a cycle of 12L:12D. Bedbugs were fed weekly using the protocol of Hase (1930). We used individuals from six large stock populations (>1000 individuals) of different origins (arbitrarily called *A, B, C, D, E, F*), which were maintained in the laboratory for different amounts of time. *A* is of unknown origin in the wild but has been maintained at the Universities of Bayreuth and Sheffield for >12 years and before that for >40 years at the London School of Hygiene and Tropical Medicine, *B* was collected in London in 2006, *C* and *D* in Nairobi in 2008, *E* in Budapest in 2010, and *F* was obtained from Bayer Environmental Science (Monheim, Germany) and maintained at the Universities of Bayreuth and Sheffield for >5 years. Over 1 year in the laboratory, bedbugs go through roughly seven generations.

All individuals in the following experiments were virgins to begin with. Last‐instar nymphs from each population were isolated and placed individually into 96‐well titer plates provided with a filter paper disk. Upon eclosion, males were collected and randomly assigned to their respective feeding regime, while females were randomly assigned to their respective mating treatment. We used pronotum width of males and females as a measure of body size because this character does not change with feeding status.

### Feeding regimes

From each population, male bedbugs were assigned to three feeding regimes: (1) fully fed, (2) half‐fed and (3) unfed. To define a feeding time for the half‐fed regime, we measured the feeding time for completing a full bloodmeal of 20 unfed males per population (*n* = 120). From this, we calculated a mean feeding time for each population (Figure S1). The mean of those population means (207 ± 12 SD) was divided by two and rounded down to 100 sec to get the half‐feeding time over all populations. All males receiving a bloodmeal were fed twice, once right at the start of the experiment, that is right after eclosion, and once the following week. Female bedbugs were fed regularly every week. All bedbugs were kept in pots provided with a filter paper grouped according to population, sex and regime.

### Effect of food restriction on sperm and seminal fluid production

The genital tract of male bedbugs consists of a pair of testes from which pairs of sperm vesicles descend to the ejaculatory pump (Usinger 1966). Next to each of the sperm vesicles, a reservoir filled with the transparent seminal fluid from the accessory glands is connected to the ejaculatory duct (Reinhardt et al. 2011). To follow sperm and seminal fluid production over time, the volumes of sperm vesicle pairs and the seminal fluid reservoirs were measured weekly for six consecutive weeks after hatching to adulthood by dissecting 30 (resp. 20 for the unfed regime, *n* = 480) males per population and feeding regime (five individuals per week, treatment and population). The first measurement took place 24 h after the first feeding and the second directly before the second feeding. For this purpose, the reproductive tract was dissected out and placed in phosphate‐buffered saline (PBS; 500 mL distilled water, 4.37 g NaCl, 0.71 g Na_2_HPO_4_) under a standard coverslip bridge preparation of defined height to measure sperm vesicle volume and seminal fluid vesicle volume (for details see Supporting Information; Otti et al. 2009 and Reinhardt et al. 2011). Individuals from the different feeding regimes were processed in a random fashion.

### Analysis of sperm and seminal fluid production

All statistical analyses were performed using R 3.1.0 (R Core Team, 2014) and the packages *car* (Fox and Weisberg 2010), *lme4* (Bates et al. 2014) *lmerTest* (Kuznetsova et al. 2014) and *multcomp* (Hothorn et al. 2008). As the dissection of all vesicles was not always successful, we only included males in the analysis for which we could define all vesicle volumes (*n* = 401). For the analysis of vesicle volume changes over time and the effect of the feeding regime, we fitted MANCOVAs (using Pillai's trace) with both volumes as a combined response variable using the *cbind()* function. The reason for this was that both vesicle volumes were measured in the same male and that they were significantly positively correlated (Pearson's correlation: *r* ^2^ = 0.67, *t* = 18.173, df = 399, *P* < 0.001). We fitted pronotum size as a covariate as it differed between populations (ANOVA: *F*_5,393_ = 48.979, *P* < 0.001). Pronotum size did not differ between feeding regimes (ANOVA: *F*_2,393_ = 1.237, *P* = 0.29), and therefore, body size was not linked to vesicle volume. As main effects, we fitted week and feeding regime and their interaction term to this combined response variable. Population was added as a random factor to account for male origin. In addition, ANOVAs were run for each response variable separately. The *P* values for the MANCOVA and these ANOVAS were corrected after Bonferroni to account for multiple testing (corrected α‐level = 0.05/3).

### Effect of food restriction on egg production

To investigate whether the effect of food restriction on ejaculate production translated into reduced female fecundity, 30 virgin females and 30 virgin males from five different populations, that is *A, B, C, E* and *F*, were reared. The virgin males were randomly assigned to one of the three feeding regimes (see “Feeding regimes”) before mating took place. For one‐half of the females, we interrupted the copulations after 60 sec to standardize ejaculate transfer (for details see Supporting Information), and for the other half of females, the males were allowed to mate with them for as long as they wanted. In addition, for the full mating treatment group we recorded the copulation duration to investigate potential differences in copulation duration between the feeding regimes. Only individuals from the same population were mated to each other. All matings (*n* = 150) were conducted 1 week after the second male feeding occurred. After mating, females were fed weekly and kept individually in vials provided with filter paper for housing and egg laying. Egg numbers were counted weekly by replacing the filter papers. We recorded the number of viable and inviable eggs, the latter being easily distinguished by their brown color and the lack of red eyes normally visible through the eggshell in viable eggs (Reinhardt et al. 2009a). Eggs were counted over a period of 8 weeks.

### Analysis of egg production

The main effects of mating treatment, feeding regime and their interaction term were fitted using linear mixed‐effect models (LME) on total egg numbers as the response variable. Population was fitted as a random effect. Among fully mated females, the total egg number was significantly positively correlated with copulation duration (Pearson's correlation: *r* ^2^ = 0.395, *t* = 3.597, df = 70, *I* < 0.001). In addition, the copulation duration differed significantly between feeding regimes (LME with box–cox transformed data (*λ* = −0.75): *F*_2,65_ = 6.32, *P* < 0.01), with fully fed males copulating longest (mean ± SD: 193.7 ± 32.5 sec) followed by half‐fed males (157.4 ± 28.2 sec) and then unfed males (142.5 ± 24.7 sec) (multiple comparisons using *multcomp* package (Hothorn et al. 2008) with *P* values adjusted according to Westfall (Bretz et al. 2010): fully fed vs. half‐fed: *P* < 0.01; fully fed versus unfed: *P* < 0.01; half‐fed vs. unfed: *P* = 0.50) (Table 1). In a second step, the total number of eggs produced from full matings was analyzed separately with copulation duration fitted as a random effect in addition to population. We checked for normality and homogeneity by visually inspecting residual versus fitted plots and the *qqnorm()* function. If transformation was needed, we fitted generalized linear models (GLMs) and used the *boxcox()* function for finding the appropriate transformation parameter lambda, that is *λ*.

**Table 1:** Mean total egg number and the mean number of inviable eggs for both mating treatments and each feeding regime. In addition, the mean copulation duration in seconds is given for the full matings and each feeding regime. First population means were calculated for each treatment combination and then the overall means and standard deviations

Male feeding regime	*n*	60‐sec mating	*n*	Full mating	Copulation duration in seconds
Fully fed	25	99 ± 16	25	107 ± 16	193.7 ± 32.5
Inviable eggs		12 ± 3		6 ± 5	
Half‐fed	25	105 ± 22	25	114 ± 21	157.4 ± 28.2
Inviable eggs		11 ± 2		10 ± 12	
Unfed	25	103 ± 13	25	98 ± 29	142.5 ± 24.7
Inviable eggs		12 ± 9		6 ± 8	

For the analysis of the proportion of inviable eggs, we used the *cbind()* function in R to combine inviable and fertile eggs as a response variable. With this response variable, we then fitted a generalized linear mixed‐effects model (GLME) with binomial distribution on mating treatment, feeding regime and their interaction term with population as a random effect. In an additional analysis, we ran the same model for only the fully mated females with population and copulation duration as a random effect.

### Effect of food restriction on male mating behavior

We investigated the effect of food restriction on male mating behavior by comparing the mating rate and the ability to mate between males from the different feeding regimes. The ability to mate we defined by measuring the time a male takes to mount a female and the copulation duration immediately after applying the feeding regimes. The time taken to mount a female served as a surrogate for mating propensity. In bedbugs, feeding dramatically changes body shape as they take up roughly ten times their body weight in liquid blood during a full meal (Reinhardt et al. 2003). To successfully mate, bedbug males need to bend the end of their abdomen to bring the intromittent organ into place for traumatic insemination. We hypothesize that males could have difficulties to mate just after feeding due to the ingested blood, which might make the bending of the abdomen harder.

#### Mating rate

We reared 70 virgin males and randomly assigned them to the three feeding regimes (see “Feeding regimes”). Due to logistic reasons and time‐consuming observations, we used only population *A* for this experiment. In addition, virgin females were produced in abundance for matings. Matings were conducted in small plastic Petri dishes (diameter 55 mm) 1 week after the second feeding of males, that is 2 weeks after eclosion, to ensure a similar flat body shape. Males were allowed to mate to as many females as they wanted within 1 h and copulation duration was standardized to 60 sec. After each 60 sec, mating a male was presented with a new, virgin female. The mating rate was calculated as the number of standardized matings of a focal male in 1 h (see also Reinhardt et al. 2011). Before mating females were fully fed, as this reduces female resistance to mating and gives males complete control over copulation (Reinhardt et al. 2009b).

#### Mating propensity and copulation duration

In the previous experiments, all males had the same flattened shape at the moment of mating. Here, we tested whether feeding, that is the different body shapes after imbibing different meal sizes, has an immediate effect on mating behavior. For this, five virgin males from each of the three feeding regimes from each of our six populations, that is *A*,* B*,* C*,* D*,* E* and *F*, were mated to a fully fed, virgin female of the same population. All matings were conducted 1 week after eclosion to adulthood of both sexes. All males were fed once, immediately before the mating, and were allowed to fully mate females. We recorded the time a male needed to mount a female, that is the mating propensity, and upon successfully mounting the time a male mated a female, that is the copulation duration.

### Analysis of male mating behavior

#### Mating rate

The main effect of feeding regime on mating rate was analyzed fitting a GLM with Gaussian distribution.

#### Mating propensity and copulation duration

We fitted an LME with the time a male needed to mount a female, that is the mating propensity, and copulation durations, respectively, as a response variable and feeding regime as a fixed factor. For both models, population was fitted as a random effect. Mating propensity was not related to copulation duration (Pearson's correlation: *r* ^2^ = 0.14, *t* = 1.405, df = 88, *P* = 0.16).

If feeding regime had a significant effect on the given response variable, we ran multiple comparisons using the *multcomp* package (Hothorn et al. 2008) to identify differences among feeding regimes adjusting *P* values according to the method by Westfall (Bretz et al. 2010). We checked for normality and homogeneity by visually inspecting residual versus fitted plots and the *qqnorm()* function. If transformation was needed, we fitted GLMs and used the *boxcox()* function for finding the appropriate transformation parameter lambda, that is *λ*.

## Results

### Effect of food restriction on sperm and seminal fluid production

#### Sperm and seminal fluid vesicles volumes combined

Body size had a significant effect on vesicle volumes (MANCOVA with Bonferroni‐corrected *P* value: Pillai's trace = 0.109, *F*_1,401_ = 22.998, *P* < 0.001). In all three feeding regimes, the vesicle volume changed significantly over time (MANCOVA with Bonferroni‐corrected *P* value: Pillai's trace = 1.036, *F*_5,401_ = 84.003, *P* < 0.001) and volumes significantly differed between feeding regimes (MANCOVA with Bonferroni‐corrected *P* value: Pillai's trace = 0.455, *F*_2,401_ = 55.440, *P* < 0.001) with fully fed males reaching the largest volumes. The three feeding regimes significantly differed in the extent of vesicle volume change over time (MANCOVA with Bonferroni‐corrected *P* value: week × feeding regime: Pillai's trace = 0.339, *F*_10,401_ = 7.686, *P* < 0.001).

#### Sperm vesicle volumes

**Figure 1:**
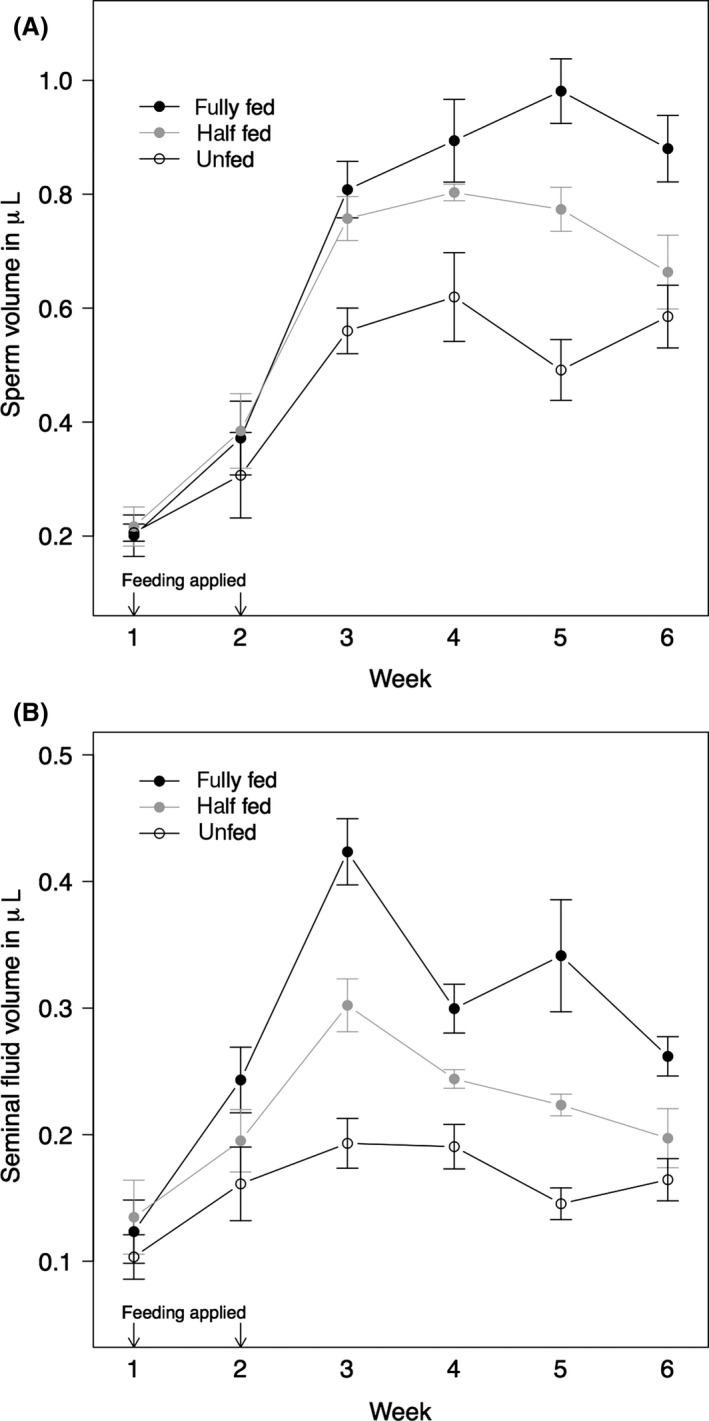
Pattern of A) sperm and B) seminal fluid production for the three feeding regimes over a 6‐week period: fully fed (black circles), half‐fed (gray circles) and unfed (open circles). Arrows indicate time of feeding. Sperm vesicle and seminal fluid vesicle volumes are given in microliters and represent averaged population means for each feeding regime (i.e., first means were calculated for each population and then the overall means and the standard errors for each feeding regime). Error bars represent one standard error.

Body size had a significant effect on sperm vesicle volumes (ANCOVA with Bonferroni‐corrected *P* value: *F*_1,401_ = 26.245, *P* < 0.001). In all three feeding regimes, the sperm vesicle volume changed significantly over time (ANCOVA with Bonferroni‐corrected *P* value: *F*_5,401_ = 212.923, *P* < 0.001). Volumes increased up to week four when the unfed and the half‐fed bedbugs reached their maximum (Fig. 1A). The difference between feeding regimes was largest in week five, when the sperm vesicles of fully fed males reached their maximum (Fig. 1A). One week after reaching, their maximum sperm vesicle volumes decreased in fully and half‐fed males. Feeding regimes significantly affected sperm production (ANCOVA with Bonferroni‐corrected *P* value: *F*_2,401_ = 67.626, *P* < 0.001). Fully fed individuals reached the highest maximum of sperm volume (mean ± SD: 0.98 ± 0.14 *μ*L), followed by half‐fed males (0.80 ± 0.04 *μ*L) and unfed males with the lowest maximum sperm volume (0.62 ± 0.19 *μ*L). Sperm production over time significantly depended on the feeding regime (ANCOVA with Bonferroni‐corrected *P* value: week × feeding regime: *F*_10,401_ = 8.651, *P* < 0.001).

#### Seminal fluid vesicle volumes

The seminal fluid vesicle volume showed a similar picture to the sperm vesicle volume except for all feeding regimes reaching their individual maximum at week three and a decrease after week four (Fig. 1B). Body size had a significant effect on seminal fluid vesicle volumes (ANCOVA with Bonferroni‐corrected *P* value: *F*_1,401_ = 33.133, *P* < 0.001). In all three feeding regimes, the seminal fluid vesicle volume changed significantly over time (ANCOVA with Bonferroni‐corrected *P* value: *F*_5,401_ = 89.787, *P* < 0.001). The difference in vesicle volumes between feeding regimes was largest in week three (Fig. 1B), and feeding regimes significantly influenced seminal fluid production (ANCOVA with Bonferroni‐corrected *P* value: *F*_2,401_ = 126.407, *P* < 0.001). Fully fed individuals reached the highest maximum of seminal fluid volume (0.42 ± 0.06 *μ*L), followed by half‐fed males (0.30 ± 0.05 *μ*L) and unfed males with the lowest maximum seminal fluid volume (0.19 ± 0.04 *μ*L). The three feeding regimes significantly differed in the extent of vesicle volume change over time (ANCOVA with Bonferroni‐corrected *P* value: week × feeding regime: *F*_10,401_ = 9.537, *P* < 0.001).

### Effect of food restriction on egg production

Neither the interaction term, nor the mating treatment or the feeding regime alone affected the total number of eggs (LME with box–cox transformed data (*λ* = 2.5): mating treatment × feeding regime: *F*_2,140_ = 0.689, *P* = 0.50; mating treatment: *F*_1,144_ = 0.919, *P* = 0.34; feeding regime: *F*_2,142_ = 0.859, *P* = 0.43) (Table 1). The random effect of population explained a significant part of the variation in total egg numbers (LME with box–cox transformed data (*λ* = 2.5): *χ* ^2^ = 11.47, df = 1, *P* < 0.001). Among fully mated females, the feeding regime did not affect the total number of eggs laid (LME with box–cox transformed data [*λ* = 2.5]: *F*_2,65_ = 0.950, *P* = 0.39), whereas the random‐effects population and copulation duration explained significant parts of the variation in total egg numbers (LME with box–cox transformed data [*λ* = 2.5]: population: *χ* ^2^ = 11.18, df = 1, *P* < 0.001; copulation duration: *χ* ^2^ = 75.52, df = 1, *P* < 0.001).

Fully mated females produced a significantly smaller proportion of inviable eggs than females mated for 60 sec (LME with binomial distribution: *χ* ^2^ = 95.066, df = 1, *P* < 0.001), and this depended on the feeding treatment (LME with binomial distribution: mating treatment × feeding regime: *χ* ^2^ = 14.496, df = 2, *P* < 0.001) (Table 1). Females mated with half‐fed males showed the smallest difference in the proportion of inviable eggs between the mating treatments, whereas the fully fed and unfed males had a similar decrease in the proportion of inviable eggs from the 60‐sec mating to the full mating. Proportion of inviable eggs did not differ between feeding regimes (LME with binomial distribution: *χ* ^2^ = 5.462, df = 2, *P* = 0.07).

### Effect of food restriction on male mating behavior

#### Mating rate

**Figure 2:**
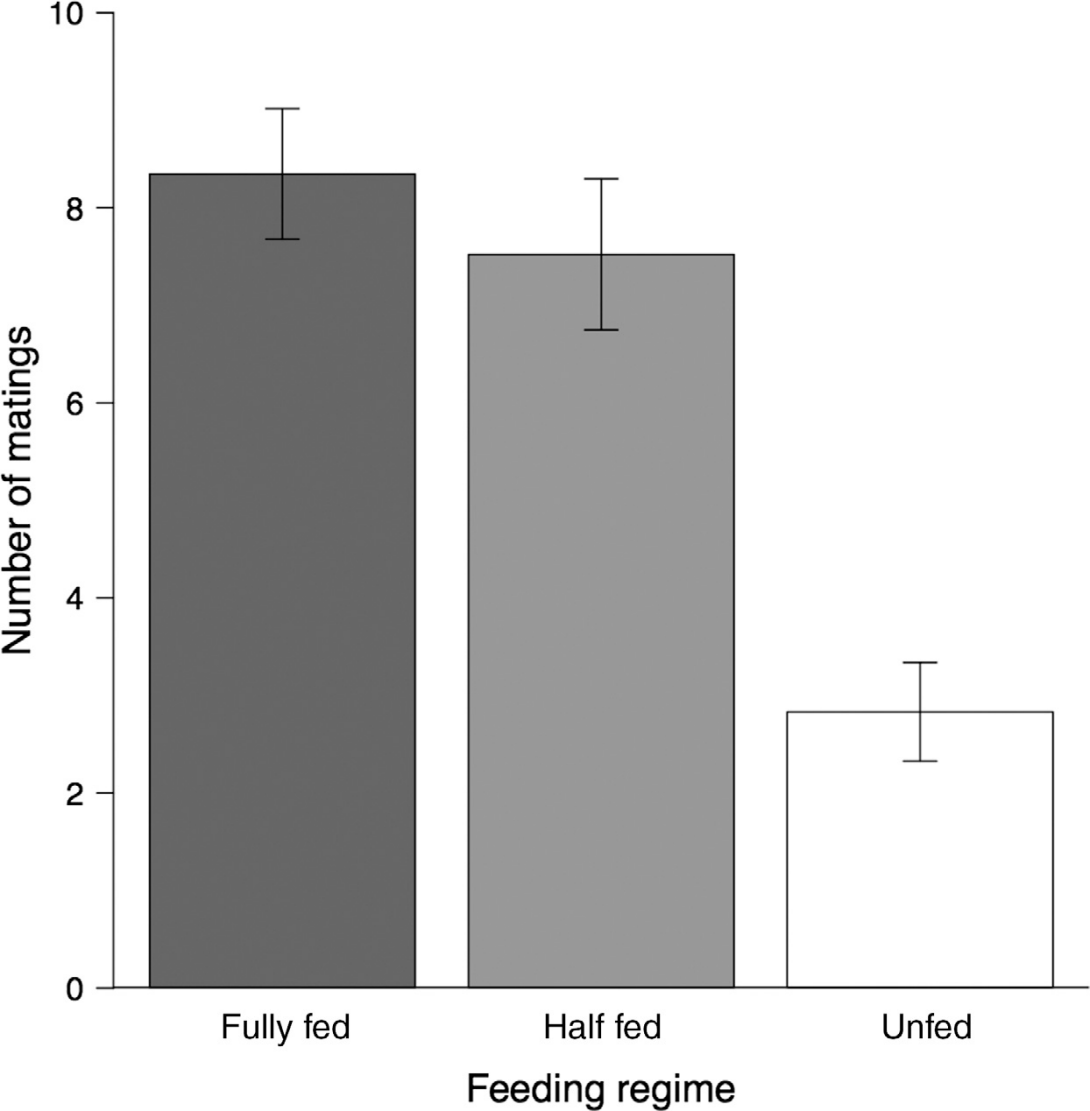
Mean number of 60‐sec matings in 1 h of males from the three feeding regimes 1 week after last feeding: fully fed (dark gray bar), half‐fed (light gray bar) and unfed (white bar). Error bars represent one standard error.

Mating rate significantly differed between feeding regimes (GLM with Gaussian distribution: *F*_2,69_ = 20.844, *P* < 0.001). Fully fed males had the highest mating rate (mean ± SD: 8.3 ± 3.2 60‐sec matings), followed by the half‐fed males (7.5 ± 3.7 60‐sec matings) and the unfed males (2.8 ± 2.5 60‐sec matings) (Fig. 2). Fully fed and half‐fed males had significantly higher mating rates than unfed males (Tukey's comparisons: fully fed vs. half‐fed: *P* = 0.38, fully fed vs. unfed: *P* < 0.001, half‐fed vs. unfed: *P* < 0.001).

#### Mating propensity and copulation duration

**Figure 3:**
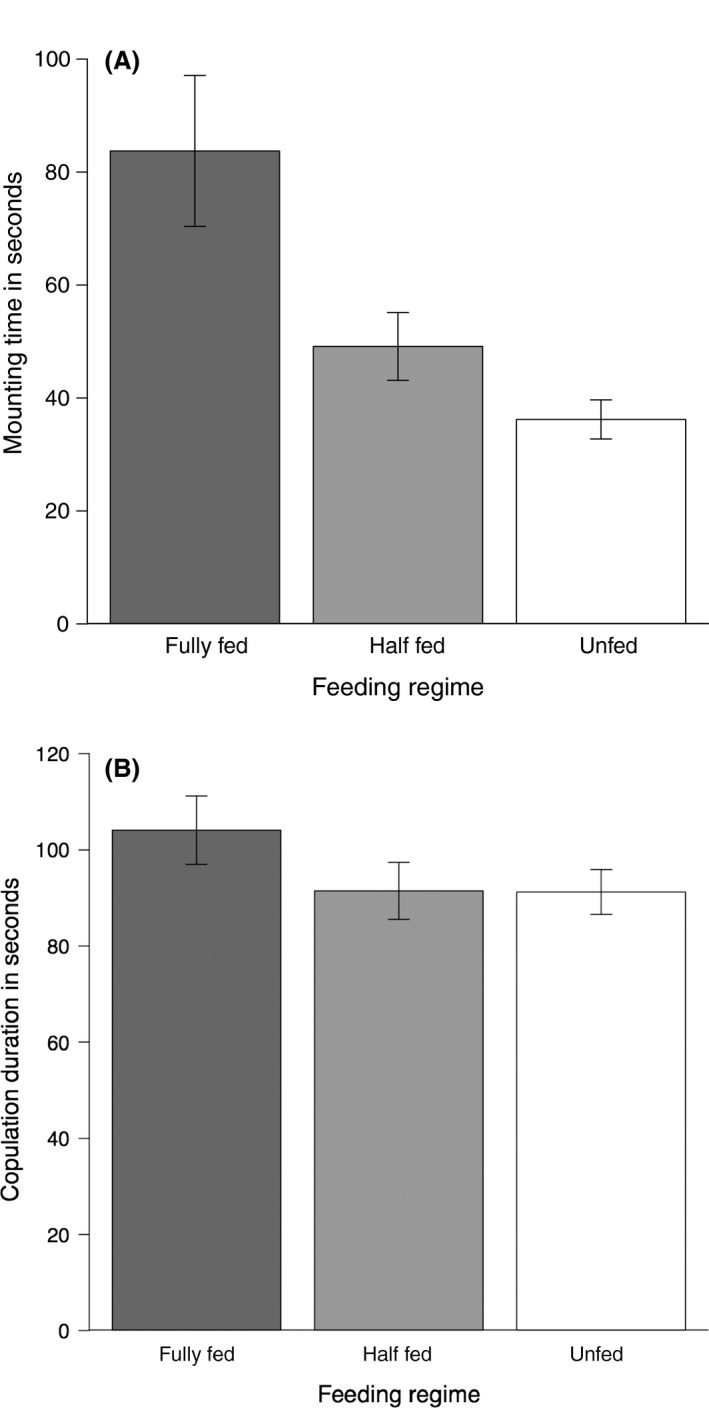
(A) Mean mounting time a surrogate for mating propensity and B) mean copulation duration in seconds of males from the three feeding regimes immediately after feeding: fully fed (dark gray bar), half‐fed (light gray bar) and unfed (white bar). Means are averaged population means, and the error bars represent one standard error.

The time a male needed to mount a female, that is the mating propensity, significantly differed between the three feeding regimes (LME with box–cox transformed data (*λ* = −0.05): *F*_2,87_ = 8.12, *P* < 0.001), with fully fed males being the slowest to mount a female (83.7 ± 32.7 sec), followed by the half‐fed males (49.1 ± 14.7 sec) and then the unfed males, which mounted females fastest (36.2 ± 8.5 sec) (Fig. 3A). Fully fed males were significantly slower than half‐fed and unfed males to mount a female (Tukey's comparisons: fully fed vs. half‐fed: *P* < 0.01, fully fed vs. unfed: *P* < 0.001, half‐fed vs. unfed: *P* = 0.17). Males from all feeding regimes copulated for a similar amount of time (LME with box–cox transformed data (*λ* = −0.32): *F*_2,82_ = 2.250, *P* = 0.11) (Fig. 3B).

## Discussion

We showed that meal size defines how much sperm and seminal fluid male bedbugs can produce, that is ejaculate production is condition dependent. The consequences of this condition dependency in males had no effect on female reproductive output in a single mating context. Most likely unfed and half‐fed males still had enough sperm for this one mating or the females could compensate for differences in sperm number or function. That single mated females laid a similar number of eggs among the male feeding regimes suggests that food restriction did not affect the quality of the ejaculate. In a multiple mated context, males could sustain mating for longer when fully fed, indicating that male mating rate is condition dependent and thereby reduces male fitness.

### Effect of food restriction on sperm and seminal fluid production

Similar to other studies, we have found that food positively affected ejaculate production (O'Dea et al. 2014). In lizards, high‐condition males produced more sperm and differed in sperm morphology in comparison with low‐condition males (Kahrl and Cox 2015). In our feeding treatments, male vesicles showed a continuous increase in volumes, with vesicles of fully fed males reaching the highest measured volume (Fig. 1A and B). In each feeding treatment, males reached the maximum vesicle volume at different time points, showing the rate of production for both ejaculate components is linked to the amount of food ingested. Both vesicle volumes declined after the initial production phase, most likely due to the fact that feeding was stopped after two feeding events. If virgin males are continuously fed the sperm, seminal fluid vesicles increase linearly until a certain point where they stay more or less the same (Reinhardt et al. 2011). Sperm and seminal fluid proteins might probably be reabsorbed if males stop feeding to prevent a potential negative effect of old sperm (Reinhardt 2007; Pizzari et al. 2008; Otti et al. 2015). Seminal fluid volume declined faster than sperm number, indicating that seminal fluid is likely the limiting factor for mating and not sperm, as previously suggested by Reinhardt et al. (2011). Kahrl and Cox (2015) hypothesize that the change in sperm morphology and sperm number might be a driver of condition‐dependent fertilization success. We suggest that condition‐dependent ejaculate production might be one mechanism leading to condition‐dependent reproductive success found in several studies (Zikovitz and Agrawal 2013; Janicke and Chapuis 2016). Taking this one step further, if variation in food availability defines the variation in available ejaculate, that is defines variation in sperm number, male feeding status might even affect sperm competition outcomes (Fricke et al. 2008; Zikovitz and Agrawal 2013).

### Effect of food restriction on male fitness

Food restriction seems to induce a fitness cost in terms of the number of copulations a male can perform rather than the number of offspring produced from a single mating. This indicates that poorly fed males are not just universally inferior, despite the reduced ejaculate component production, but that reduced fitness becomes apparent in a competitive scenario. Males having fewer sperm and less seminal fluid available due to low food availability did not have fewer offspring when mated to one female. Interestingly, even though unfed males copulated for a shorter time in the full matings, their female mates did lay a similar amount of eggs as the females mated to males from the other feeding regimes. Also the proportion of inviable eggs was higher in the 60‐sec mating than in the full mating. As bedbug males transfer ejaculate at a linear rate during mating (Siva‐Jothy and Stutt 2003), we assume that the ejaculate of unfed males was somehow of better quality or contained more sperm relative to seminal fluid. Unfed males might have had fewer dysfunctional or dead sperm (Reinhardt 2007), or their seminal fluid was composed of agents that increase fertilization success (Poiani 2006). The equal proportions of inviable eggs laid by females among feeding regimes represent another indicator for high ejaculate quality in unfed males relative to males from the other feeding regimes (Reinhardt et al. 2009a). Similar to ladybird beetles, where low‐condition males transfer more sperm relative to other ejaculate components to females than high‐condition males (Perry and Rowe 2010), unfed bedbug males could also have transferred more sperm relative to seminal fluid than half‐fed or fully fed males. As we did not measure ejaculate transfer or sperm viability, these hypotheses remain to be tested. Nevertheless, our findings confirm that adult male bedbugs can produce enough ejaculate with good‐quality sperm for at least one single mating without any food (Buxton 1930).

The mating rate significantly increased with food availability. With larger meals, males were able to achieve a higher mating rate, that is higher fitness, than males with smaller meals or none at all. As feeding regulates ejaculate production in a very direct manner, we can rule out ejaculate replenishment between the matings in our experiment, and therefore, we predict that ejaculate reserves decreased over successive matings similar to other insects (Damiens and Boivin 2005). From this, we conclude that food‐restricted males have reduced reproductive fitness, as their number of potential mates is limited by the available ejaculate. Under starvation or restricted access to food mating costs increased in male butterflies *Callophrys xami* (Cordero 2000) and male red flour beetles *Tribolium castaneum* (Sbilordo et al. 2011), suggesting that balancing the investment in reproduction under food‐limited conditions is more difficult (Scharf et al. 2013). As food restriction significantly reduced available ejaculate size and consequently male mating rate, our results are consistent with Reinhardt et al. (2011) showing that ejaculate depletion defines the mating rate. Reinhardt et al. (2011) calculated that it takes roughly seven matings for sperm to be depleted in comparison with roughly four matings, after which seminal fluid is depleted. Our study found that unfed males could perform almost three matings. Therefore, condition‐dependent ejaculate production translated into a condition‐dependent male mating rate reducing male reproductive fitness. In a competitive scenario, for males that are low on ejaculate, the fitness loss will potentially be amplified. In future, also evidence is needed if such an immediate reduction in mating rate due to low food availability could be compensated by males, at times when food is more abundant again.

On the one hand, large meals significantly reduced mating propensity, suggesting that under male–male competition fully fed males might suffer from reduced fitness. In flies, mating propensity is positively related to temperature (Cook 1994) and seems to depend on the body size relationship between mating partners (Churchill‐Stanland et al. 1986; Aluja et al. 2009). In addition, male diet was shown to impact on female mating latency depending on the females body size (Fricke et al. 2008; Aluja et al. 2009). Large females seem not to differentiate between male nutritional status, whereas small females mated quicker with males on a low‐quality diet (Aluja et al. 2009). Although many studies exist that aimed to understand factors affecting mating propensity including nutrition, our study seems to be the first to measure an immediate effect of feeding and meal size on the ability to mate in males. On the other hand, meal size did not affect copulation duration, suggesting that once a female has been successfully mounted the enlarged abdomen seems not to hinder copulation itself.

Nevertheless, males face a similar problem as females, which cannot resist mating after a full bloodmeal (Reinhardt et al. 2009b). For males, a large meal seems to reduce the ability to run after and to mount females. Assuming that unfed males have an advantage over fully fed males when competing for females, we suggest to investigate the effect of feeding status on male mating behavior in a next step. In addition, the reduced mating propensity due to feeding in bedbug males represents a short‐term effect on the ability to mate, because males will only be hindered at mounting females during the digestion of bloodmeals. In combination with the finding that male mating rate is dependent on the amount of food ingested, that is the amount of ejaculate available to a male, we suggest that males should adjust their feeding behavior according to the presence of mating partners and/or competitors to optimize feeding and mating opportunities. More explicitly, as males seem to suffer from a short‐term negative effect when taking a full bloodmeal, we hypothesize that it might be beneficial for males to strategically feed to balance a potential trade‐off between eating, that is having enough sperm, and staying thin to mount females fast.

## Conclusion

We provide new evidence that the amount of ingested food has an effect on reproductive traits in males, which translates into a condition‐dependent male mating rate. Variation in food availability can therefore affect male fitness as the number of mates is reduced under food restriction. An interesting next step would be to investigate the consequences of such an immediate mating cost for male lifetime reproductive success. In addition, it remains to be shown whether males could compensate for a short‐term fitness loss due to low food in the environment, at times when food is more abundant again. A potential trade‐off between present and future reproductive success could be mediated by food availability and at least hypothetically affect the evolution of mating strategies. It seems that natural selection influences variation in reproductive processes and therefore might impact on traits under sexual selection. For example, we could imagine that variation in ejaculate sizes induced by variation in food availability could affect sperm competition outcomes when males with different feeding levels mate with the same female. With our study, we would like to emphasize the importance of ecological context in the study of reproduction. Despite the immense importance of ecological contexts reported in reproductive medicine, studies in evolutionary and ecological research trying to understand the sources of variation in reproductive success neglect environmental factors affecting variation in reproductive traits, especially the ecological effects on sperm and the ejaculate as a whole (reviewed in Reinhardt et al. 2015).

## Conflict of Interest

None declared.

## Supporting information


**Figure S1.** Mean male feeding times in seconds for each population.

## References

[ece32073-bib-0001] Aitken, R. J. , T. B. Smith , M. S. Jobling , M. A. Baker , and G. N. De Iuliis . 2014 Oxidative stress and male reproductive health. Asian J. Androl. 16:31–38. 24369131 10.4103/1008-682X.122203 PMC3901879

[ece32073-bib-0002] Aluja, M. , J. Rull , J. Sivinski , G. Trujillo , and D. Pérez‐Staples . 2009 Male and female condition influence mating performance and sexual receptivity in two tropical fruit flies (Diptera: Tephritidae) with contrasting life histories. J. Insect Physiol. 55:1091–1098. 19666025 10.1016/j.jinsphys.2009.07.012

[ece32073-bib-0003] Andersson, M. B. 1994 Sexual selection. Princeton Univ. Press, Princeton, NJ.

[ece32073-bib-0004] Avila, F. W. , L. K. Sirot , B. A. LaFlamme , C. D. Rubinstein , and M. F. Wolfner . 2011 Insect seminal fluid proteins: identification and function. Annu. Rev. Entomol. 56:21–40. 20868282 10.1146/annurev-ento-120709-144823 PMC3925971

[ece32073-bib-0005] Bates, D. , M. Maechler , B. Bolker , and S. Walker . 2014 lme4: linear mixed‐effects models using Eigen and S4. R package version 1.1‐6. https://CRAN.R-project.org/package=lme4.

[ece32073-bib-0006] Boggs, C. L. 1992 Resource allocation: exploring connections between foraging and life history. Funct. Ecol. 6:508.

[ece32073-bib-0007] Boggs, C. L. 2009 Understanding insect life histories and senescence through a resource allocation lens. Funct. Ecol. 23:27–37.

[ece32073-bib-0008] Bretz, F. , T. Hothorn , and P. Westfall . 2010 Multiple comparisons using R. Chapman and Hall/CRC Press, London, UK.

[ece32073-bib-0009] Buxton, P. A. 1930 The biology of a blood‐sucking bug, *Rhodnius prolixus* . Trans. R Entomol. Soc. Lond 31:227–236.

[ece32073-bib-0010] Churchill‐Stanland, C. , R. Stanland , T. T. Y. Wong , N. Tanaka , D. O. McInnis , and R. V. Dowell . 1986 Size as a factor in the mating propensity of mediterranean fruit flies, *Ceratitis capitata* (Diptera: Tephritidae), in the laboratory. J. Econ. Entomol. 79:614–619.

[ece32073-bib-0011] Cook, D. F. 1994 Influence of temperature on copula duration and mating propensity in *Lucilia cuprina* Wiedemann (Diptera: Calliphoridae). Aust. J. Entomol. 33:5–8.

[ece32073-bib-0012] Cordero, C. 2000 Trade‐off between fitness components in males of the polygynous butterfly *Callophrys xami* (Lycaenidae): the effect of multiple mating on longevity. Behav. Ecol. Sociobiol. 48:458–462.

[ece32073-bib-0013] Cotton, S. , K. Fowler , and A. Pomiankowski . 2004 Do sexual ornaments demonstrate heightened condition‐dependent expression as predicted by the handicap hypothesis? Proc. Biol. Sci. 271:771–783. 15255094 10.1098/rspb.2004.2688 PMC1691662

[ece32073-bib-0500] Damiens, D. , and G. Boivin . 2005 Male reproductive strategy in *Trichogramma evanescens*: sperm production and allocation to females. Physiol Entomol 30:1365‐3032.

[ece32073-bib-0014] Dewsbury, D. 1982 Ejaculate cost and male choice. Am. Nat. 119:601–610.

[ece32073-bib-0015] Fox, J. , and S. Weisberg . 2010 An R companion to applied regression, 2nd edn SAGE Publications Inc., Thousand Oaks, CA.

[ece32073-bib-0016] Fricke, C. , A. Bretman , and T. Chapman . 2008 Adult male nutrition and reproductive success in *Drosphila melanogaster* . Evolution 62:3170–3177. 18786187 10.1111/j.1558-5646.2008.00515.x

[ece32073-bib-0017] Hase, A. 1930 Methoden der Züchtung von Wanzen, Läusen und Flöhen Pp. 591–616 *in* Kolle W., Kraus R. and Uhlenhut P., eds. Handbuch der pathogenen Mikroorganismen, 3rd edn Gustav Fischer, Jena.

[ece32073-bib-0018] Hodgson, D. J. , and D. J. Hosken . 2006 Sperm competition promotes the exploitation of rival ejaculates. J. Theor. Biol. 243:230–234. 16901507 10.1016/j.jtbi.2006.06.024

[ece32073-bib-0019] Hodin, J. 2009 She shapes events as they come: plasticity in female insect reproduction Pp. 423–521 *in* Whitman D. W. and Ananthakrishnan T. N., eds. Phenotypic plasticity of insects mechanisms and consequences. CRC Press, Boca Raton, FL.

[ece32073-bib-0020] Hothorn, T. , F. Bretz , and P. Westfall . 2008 Simultaneous inference in general parametric models. Biom. J. 50:346–363. 18481363 10.1002/bimj.200810425

[ece32073-bib-0021] Janicke, T. , and E. Chapuis . 2016 Condition dependence of male and female reproductive success: insights from a simultaneous hermaphrodite. Ecol. Evol. 6:830–841. 26865970 10.1002/ece3.1916 PMC4739575

[ece32073-bib-0022] Jensen, T. K. , A.‐M. Andersson , N. Jorgensen , A.‐G. Andersen , E. Carlsen , J. H. Petersen , et al. 2004 Body mass index in relation to semen quality and reproductive hormones among 1,558 Danish men. Fertil. Steril. 82:863–870. 15482761 10.1016/j.fertnstert.2004.03.056

[ece32073-bib-0023] Kahrl, A. M. , and R. M. Cox . 2015 Diet affects ejaculate traits in a lizard with condition‐dependent fertilisation success. Behav. Ecol. 26:1502–1511.

[ece32073-bib-0024] Kuznetsova, A. , P. B. Brockhoff , and R. H. B. Christensen . 2014 lmerTest: tests in linear mixed effects models. R package version 2.0‐20. https://cran.r-project.org/package=lmerTest

[ece32073-bib-0025] Lee, K. P. , S. J. Simpson , F. J. Clissold , R. Brooks , J. W. O. Ballard , P. W. Taylor , et al. 2008 Lifespan and reproduction in *Drosophila*: new insights from nutritional geometry. Proc. Natl Acad. Sci. USA 105:2498–2503. 18268352 10.1073/pnas.0710787105 PMC2268165

[ece32073-bib-0026] Morehouse, N. I. , T. Nakazawa , C. M. Booher , P. D. Jeyasingh , and M. D. Hall . 2010 Sex in a material world: why the study of sexual reproduction and sex‐specific traits should become more nutritionally explicit. Oikos 119:766–778.

[ece32073-bib-0027] O'Dea, R. E. , M. D. Jennions , and M. L. Head . 2014 Male body size and condition affects sperm number and production rates in mosquitofish, *Gambusia holbrooki* . J. Evol. Biol. 27:2739–2744. 25403851 10.1111/jeb.12534

[ece32073-bib-0028] Otti, O. , R. A. Naylor , M. T. Siva‐Jothy , and K. Reinhardt . 2009 Bacteriolytic activity in the ejaculate of an insect. Am. Nat. 174:292–295. 19548839 10.1086/600099

[ece32073-bib-0029] Otti, O. , P. R. Johnston , G. J. Horsburgh , J. Galindo , and K. Reinhardt . 2015 Female transcriptomic response to male genetic and nongenetic ejaculate variation. Behav. Ecol. 26:681–688.

[ece32073-bib-0030] Perry, J. C. , and L. Rowe . 2010 Condition‐dependent ejaculate size and composition in a ladybird beetle. Proc. Biol. Sci. 277:3639–3647. 20573622 10.1098/rspb.2010.0810 PMC2982242

[ece32073-bib-0031] Pizzari, T. , R. Dean , A. Pacey , H. Moore , and M. B. Bonsall . 2008 The evolutionary ecology of pre‐ and post‐meiotic sperm senescence. Trends Ecol. Evol. 23:131–140. 18280006 10.1016/j.tree.2007.12.003

[ece32073-bib-0032] Poiani, A. 2006 Complexity of seminal fluid: a review. Behav. Ecol. Sociobiol. 60:289–310.

[ece32073-bib-0033] R Core Team . 2014 R: a language and environment for statistical computing. R Foundation for Statistical Computing, Vienna, Austria.

[ece32073-bib-0034] Reinhardt, K. 2007 Evolutionary consequences of sperm cell aging. Q. Rev. Biol. 82:375–393. 18217528 10.1086/522811

[ece32073-bib-0035] Reinhardt, K. , and M. T. Siva‐Jothy . 2007 Biology of the bed bugs (Cimicidae). Annu. Rev. Entomol. 52:351–374. 16968204 10.1146/annurev.ento.52.040306.133913

[ece32073-bib-0036] Reinhardt, K. , R. Naylor , and M. T. Siva‐Jothy . 2003 Reducing a cost of traumatic insemination: female bedbugs evolve a unique organ. Proc. Biol. Sci. 270:2371–2375. 14667353 10.1098/rspb.2003.2515 PMC1691512

[ece32073-bib-0037] Reinhardt, K. , R. A. Naylor , and M. T. Siva‐Jothy . 2009a Ejaculate components delay reproductive senescence while elevating female reproductive rate in an insect. Proc. Natl Acad. Sci. USA 106:21743–21747. 19996174 10.1073/pnas.0905347106 PMC2799855

[ece32073-bib-0038] Reinhardt, K. , R. A. Naylor , and M. T. Siva‐Jothy . 2009b Situation exploitation: higher male mating success when female resistance is reduced by feeding. Evolution 63:29–39. 18752607 10.1111/j.1558-5646.2008.00502.x

[ece32073-bib-0039] Reinhardt, K. , R. Naylor , and M. T. Siva‐Jothy . 2011 Male mating rate is constrained by seminal fluid availability in bedbugs, *Cimex lectularius* . PLoS One 6:e22082. 21779378 10.1371/journal.pone.0022082 PMC3136940

[ece32073-bib-0040] Reinhardt, K. , R. Dobler , and J. Abbott . 2015 An ecology of sperm: sperm diversification by natural selection. Annu. Rev. Ecol. Evol. Syst. 46:435–459.

[ece32073-bib-0041] Sbilordo, S. H. , V. M. Grazer , M. Demont , and O. Y. Martin . 2011 Impacts of starvation on male reproductive success in *Tribolium castaneum* . Evol. Ecol. Res. 13:347–359.

[ece32073-bib-0042] Scharf, I. , F. Peter , and O. Y. Martin . 2013 Reproductive trade‐offs and direct costs for males in arthropods. Evol. Biol. 40:169–184.

[ece32073-bib-0043] Simmons, L. W. 2001 Sperm competition and its evolutionary consequences in the insects, Monographs in Behavior and Ecology. Princeton Univ. Press, Princeton, NJ.

[ece32073-bib-0044] Sirot, L. K. , M. F. Wolfner , and S. Wigby . 2011 Protein‐specific manipulation of ejaculate composition in response to female mating status in *Drosophila melanogaster* . Proc. Natl Acad. Sci. USA 108:9922–9926. 21628597 10.1073/pnas.1100905108 PMC3116428

[ece32073-bib-0045] Siva‐Jothy, M. T. , and A. D. Stutt . 2003 A matter of taste: direct detection of female mating status in the bedbug. Proc. Biol. Sci. 270:649–652. 12769466 10.1098/rspb.2002.2260 PMC1691276

[ece32073-bib-0046] Sofikitis, N. , I. Miyagawa , D. Dimitriadis , P. Zavos , S. Sikka , and W. Hellstrom . 1995 Effects of smoking on testicular function, semen quality and sperm fertilizing capacity. J. Urol. 154:1030–1034. 7637048

[ece32073-bib-0047] Stutt, A. D. , and M. T. Siva‐Jothy . 2001 Traumatic insemination and sexual conflict in the bed bug *Cimex lectularius* . Proc. Natl Acad. Sci. USA 98:5683–5687. 11331783 10.1073/pnas.101440698 PMC33273

[ece32073-bib-0048] Usinger, R. L. 1966 Monograph of Cimicidae. Entomological Society of America, College Park, MD.

[ece32073-bib-0049] Wedell, N. , M. J. G. Gage , and G. A. Parker . 2002 Sperm competition, male prudence and sperm‐limited females. Trends Ecol. Evol. 17:313–320.

[ece32073-bib-0050] Zikovitz, A. E. , and A. F. Agrawal . 2013 The condition dependency of fitness in males and females: the fitness consequences of juvenile diet assessed in environments differing in key adult resources. Evolution 67:2849–2860. 24094338 10.1111/evo.12170

