# Supplementary material for: Condition‐dependent ejaculate production affects male mating behavior in the common bedbug Cimex lectularius
Source: Ecol Evol. 2016 Mar 14;6(8):2548–58. doi: 10.1002/ece3.2073 (PMC4797159; doi:10.1002/ece3.2073)
Supplement: Supplementary file 1 — Figure S1. Mean male feeding times in seconds for each population. [file ECE3-6-2548-s001.docx]

**Supporting information for Kaldun and Otti 2016**


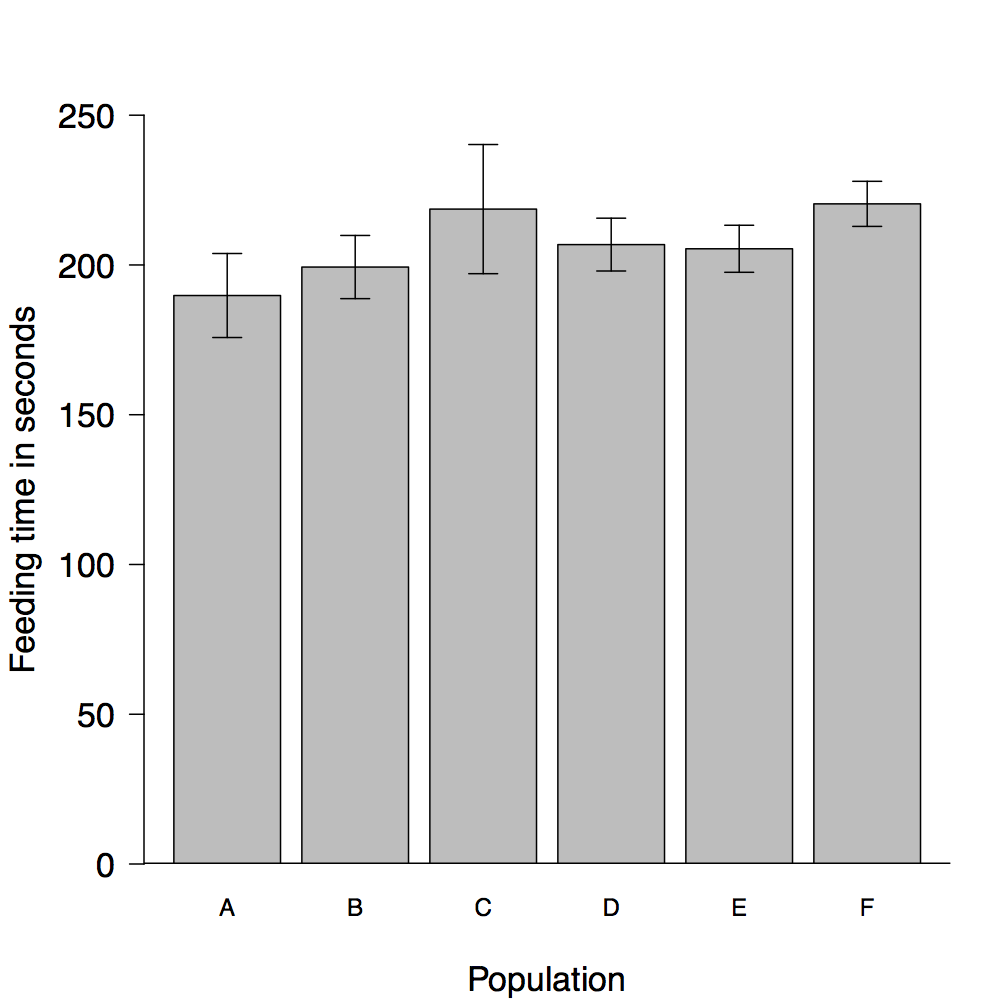


**Figure S1)** Mean male feeding times in seconds for each population. Feeding times were measured for 20 males from each population (N=120). Male feeding times did not differ between populations (ANOVA: F_5,120_=0.833, P=0.53). Overall mean feeding time was 207±12 (s.d.) seconds. Error bars represent one standard error.

**Coverslip bridge preparation**

Our standard coverslip bridge preparation is made from two stacks of coverslips (two coverslips of 18 x 18mm and ~0.3 mm thickness) glued to a microscope slide 15mm apart from each other. Between the stacks the sample is placed in 10μl of PBS and another microscope slide is used as a cover (see also Otti etal. 2009 and Reinhardt *et al.*, 2011). From this a picture of the entire male reproductive tract was taken at a defined magnification using a stereomicroscope (Leica S8 APO, Leica Microsystems, Wetzlar, Germany), a digital camera (Leica DFC290, Leica Microsystems), and the Leica Application Suite software (version 2.7.1 R1 [Build: 1384], Leica Microsystems). The area of the sperm vesicles and also the seminal fluid vesicles was measured from these images using the software ImageJ (version 1.44p, National Institutes of Health, USA). For calibration of the area we used graph paper. As the area was linearly proportional to volume we calculated the vesicle volumes in μl from the area taken up by 1μl of PBS under the constant coverslip bridge. Individuals from the different feeding regimes were processed in a random fashion.

**Bedbug matings**

In bedbugs copulation duration and ejaculate transfer are related to each other (Siva-Jothy & Stutt, 2003). This allows to standardise ejaculate transfer with fixed copulation durations and to compare it to a natural copulation duration chosen by a given male. For the matings one individual from each sex was put in small plastic petri dishes (diameter 55 mm) provided with filter paper and the complete copulation sequence was observed.

**References**

Otti, O., Naylor, R.A., Siva-Jothy, M.T. & Reinhardt, K. 2009. Bacteriolytic activity in the ejaculate of an insect. *Am Nat* **174**: 292–295.

Reinhardt, K., Naylor, R. & Siva-Jothy, M.T. 2011. Male mating rate is constrained by seminal fluid availability in bedbugs, *Cimex lectularius*. *PLoS One* **6**: e22082.

Siva-Jothy, M.T. & Stutt, A.D. 2003. A matter of taste: direct detection of female mating status in the bedbug. *Proc R Soc Lond B* **270**: 649–652.
